# Towards Early Poultry Health Prediction through Non-Invasive and Computer Vision-Based Dropping Classification

**DOI:** 10.3390/ani13193041

**Published:** 2023-09-27

**Authors:** Arnas Nakrosis, Agne Paulauskaite-Taraseviciene, Vidas Raudonis, Ignas Narusis, Valentas Gruzauskas, Romas Gruzauskas, Ingrida Lagzdinyte-Budnike

**Affiliations:** 1 Faculty of Informatics, Kaunas University of Technology, Studentu 50, 51368 Kaunas, Lithuania; arnas.nakrosis@ktu.lt (A.N.); ignas.narusis@ktu.edu (I.N.); ingrida.lagzdinyte@ktu.lt (I.L.-B.); 2 Artificial Intelligence Centre, Kaunas University of Technology, K. Barsausko 59, 51423 Kaunas, Lithuania; vidas.raudonis@ktu.lt (V.R.); valentas.gruzauskas@mif.vu.lt (V.G.); romas.gruzauskas@ktu.lt (R.G.); 3 Faculty of Electrical and Electronics, Kaunas University of Technology, Studentu 48, 51367 Kaunas, Lithuania; 4 Institute of Computer Science, Vilnius University, 08303 Vilnius, Lithuania

**Keywords:** poultry, droppings, computer vision, deep learning, segmentation, classification

## Abstract

**Simple Summary:**

The integration of artificial intelligence and advanced computer vision techniques holds significant promise for non-invasive health assessments within the poultry industry. Monitoring poultry health through droppings can provide valuable insights as alterations in texture and color may signal the presence of severe and contagious illnesses. This study, in contrast to previous research that often employed binary or limited multi-class classifications for droppings, employs image processing algorithms to categorize droppings into six distinct classes, each representing various abnormality levels, with data collected from three different poultry farms in Lithuania, including diverse litter types.

**Abstract:**

The use of artificial intelligence techniques with advanced computer vision techniques offers great potential for non-invasive health assessments in the poultry industry. Evaluating the condition of poultry by monitoring their droppings can be highly valuable as significant changes in consistency and color can be indicators of serious and infectious diseases. While most studies have prioritized the classification of droppings into two categories (normal and abnormal), with some relevant studies dealing with up to five categories, this investigation goes a step further by employing image processing algorithms to categorize droppings into six classes, based on visual information indicating some level of abnormality. To ensure a diverse dataset, data were collected in three different poultry farms in Lithuania by capturing droppings on different types of litter. With the implementation of deep learning, the object detection rate reached 92.41% accuracy. A range of machine learning algorithms, including different deep learning architectures, has been explored and, based on the obtained results, we have proposed a comprehensive solution by combining different models for segmentation and classification purposes. The results revealed that the segmentation task achieved the highest accuracy of 0.88 in terms of the Dice coefficient employing the K-means algorithm. Meanwhile, YOLOv5 demonstrated the highest classification accuracy, achieving an ACC of 91.78%.

## 1. Introduction

Adequate production capacity to produce high quality and safe products is a key factor in the efficient operation of the poultry sector. In order to maintain the efficiency of the sector, it is necessary not only to ensure good conditions for poultry farming in line with animal welfare requirements, but also to control the technological parameters of production and to ensure the prevention of health problems in poultry in order to avoid losses at the initial stage of production. The concept of sustainable production has recently received considerable attention, with analysis of the environmental impact of poultry meat production and the development of production technologies, taking into account the European Green Deal strategy and the Food and Agriculture Organisation and the European Feed Manufacturers’ Federation strategic guidelines. These strategies focus on the reduction in odor dispersion and greenhouse gas emissions, with ammonia (NH3) [1] and hydrogen sulfide (H_2_S) as harmful gases and CO_2_, CH_4_ and N_2_O as greenhouse gases. Poultry farming also contributes to environmental pollution through the formation of volatile organic compounds (VOCs). These compounds are another category of substances that are associated with environmental pollution. The organic compounds released during the production of poultry meat contribute to the pollution of the environment by residues of macro and trace elements of these compounds. The health of a flock is greatly influenced by the nutrient uptake of feed, which, in turn, has a direct correlation with environmental pollution. Analyzing feed nutrients and health indicators requires a substantial investment of human resources as it demands the expertise of highly qualified specialists and the utilization of specialized and often costly techniques and equipment. In the agricultural sector, AI-driven technologies have the potential to push the industry forward as AI can bring many benefits to the sector, such as early prediction systems, disease identification, automated feeding and nutritional analysis, self-monitoring systems for tracking animal behavior and, in general, contributing to improving the efficiency of farm management practices by providing timely insights and alerts to farmers and/or veterinarians. Overall, harnessing the power of data analysis and intelligent decision making by taking into account information gained from various smart sensors is paving the way to a smarter and more sustainable agriculture in the future.

The deployment and use of Artificial Intelligence (AI) technology in the field of agriculture is also rapidly gaining popularity in response to the escalating global population and the corresponding increased demand for food [1,2,3]. Multiple factors such as climate change, a burgeoning population, increased food consumption and employment issues have contributed to this trend. Recognizing the urgent need for modern and more sophisticated technologies, the agricultural sector is increasingly turning to AI [3,4,5]. As a result, the role of AI in poultry farming has gained significant prominence [6]. In the poultry industry, the application of image processing technologies has produced impressive results [7,8,9,10]. However, data collection and capture commonly rely on IoT technologies.

Currently, the integration of IoT technologies into the poultry farming business in Lithuania and worldwide is relatively low. Scientific sources extensively discuss cutting-edge farming technologies that involve analyzing temperature fluctuations within the flock, examining the effects of oxidative and thermal stress on both health and productivity, exploring the development of an optimal microclimate and assessing the influence of noise levels on the well-being of birds, among other factors [11,12,13].

In broiler chicken production, the utilization of the IoT is highlighted for various purposes. One notable application involves predicting chicken health by analyzing their appearance, behavior and microclimatic parameters [14,15]. Through the collection and analysis of such information, it becomes feasible to identify the presence of particular diseases in chickens [16,17] and subsequently implement more efficient preventive measures. This enables improved disease management and control in the poultry farming industry [18]. In digital production, the chickens can be monitored in real time. This includes automated scales that send continuous data on body weight, temperature and feed intake to improve production efficiency, the welfare and health of the birds, more effective nutritional strategy, biosecurity and odor reduction. It is very important for large poultry farms as it allows them to analyze very large flocks of chickens to predict growth trends and to adjust feed production, quality, rearing conditions and the production of high quality poultry meat accordingly. Moreover, AI techniques can also be applied to DNA research, facilitating the development of disease-resistant combinations of poultry lines, rectifying defects in poultry meat and creating products with enhanced nutritional value [19,20,21,22]. However, the effective integration of these technologies into the poultry production chain is still pending.

Currently, the utilization of advanced technologies as a reliable means for controlling and preventing bird health issues in the poultry sector is not adequately effective. Inadequate preventive measures at later stages result in significant losses due to bird deaths. Increased bird morbidity is directly associated with elevated environmental pollution, which encompasses not only heightened odor and CO_2_ emissions but also the discharge of drug residues into the environment. Consequently, this leads to a decline in production quality, causing substantial losses for agricultural companies. Moreover, consumers are negatively impacted by the inferior quality and increased cost of the produce.

The poultry farming sector faces several challenges, particularly in terms of hygiene and diseases. Common ailments include Salmonella, Gumboro pullorum, Newcastle, and Coccidiosis [23]. Diagnostic laboratory procedures for these conditions are typically time consuming and performed manually, with considerable expense. For example, American laboratories (e.g., GPLN and others) charge an average of $30 for bacteriological tests on poultry feces, and the price varies based on the number of birds tested [24,25]. To detect disease and eliminate the disease source as early as possible, poultry workers must monitor individual chickens for any behavioral or physical changes [16]. Assessing the condition of chickens through monitoring their feces provides a non-invasive approach, as significant changes in consistency and color may indicate certain abnormalities prompting further investigation into potential causes like diseases or infections [26]. However, these indicators also depend on chicken feed, with greenish stools resulting from grass consumption and black stools from blackberry consumption [3].

In this study, we have implemented computer vision technologies, focusing on time-sensitive production control and the primary detection of abnormal poultry droppings, through non-invasive droppings analysis, thus ensuring the timely control of possible disease outbreaks and improved flock health. Therefore, the main objective of this research is to develop a deep learning-based approach for monitoring poultry droppings.

## 2. Related Works

The classification of poultry droppings is a critical task in poultry farming which involves categorizing droppings based on their characteristics, such as color, consistency, water content and texture. Several studies have been conducted with the objective of classifying or segmenting droppings using image analysis. But, it should be noted that there is significant variation in terms of experimental conditions, the establishment of ground truth (e.g., litter) and the number of classes used in these studies. In the simplest scenario, the classification task focuses on a binary distinction, categorizing droppings as either healthy or unhealthy [27]. However, given the increasing importance of the early detection of diseases or infections, a multi-class classification approach is becoming more relevant. For example, in a recent study [28], researchers identify the eight prevalent diseases (such as Avian influenza, Infectious bursa disease, Pullorum disease, etc.) that lead to diarrhea in chickens and highlight visual distinctive dropping characteristics associated with each disease. Additionally, the study highlights the vulnerable time periods and the level of risk associated with these diseases. Visual differences in droppings affected by the above diseases can be quite apparent when captured under controlled laboratory conditions with close-up shots of the specimens, and so on. In some investigations, fecal images are taken on a conveyor line, which can lead to very different results under realistic conditions [29]. The study encompassed five heuristic classes, with one class representing normal fecal samples and the remaining classes indicating abnormalities in terms of shape, color or a combination of both. Other authors classify droppings into three categories, “Coccidiosis”, “Health” and “Salmonella”. A high accuracy of 93.67% was achieved using the fully connected CNN model [16]. A four-class classification model has also been proposed, with one additional class, Newcastle Disease [30]. This model utilizes two deep learning architectures, namely YOLOv3 for object detection and the ResNet50 algorithm for image classification, and achieved an accuracy of 98.71%.

Various approaches can be used for this task, including unsupervised and supervised machine learning techniques. Unsupervised approaches for poultry dropping classification (more specifically clustering) do not require labelled data and rely on the intrinsic characteristics of the data itself. However, to achieve high accuracy in this task, the features extracted must be highly distinctive, and the complexity of the multi-class classification makes this task challenging. In contrast, supervised approaches rely on labeled data to train machine learning models capable of accurately classifying the droppings. It is crucial to have a substantial amount of labeled data to ensure effective training. However, it is evident that deep learning architectures exhibit the greatest potential in this context as these models are capable of automatically learning complex data patterns and performing object detection, segmentation and classification tasks.

## 3. Materials and Methods

**Figure 1:**
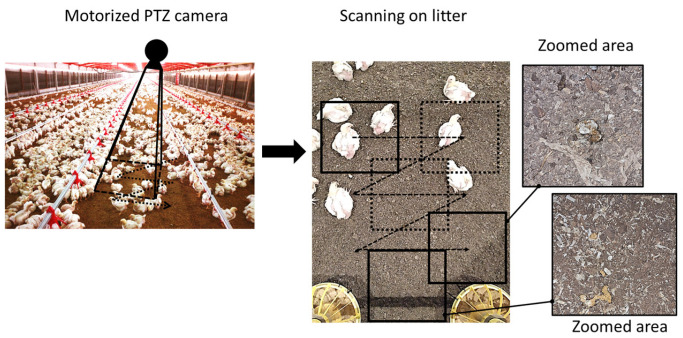
The process of gathering litter images in poultry farm.

A motorized pan-tilt-zoom (PTZ) camera, mounted at a height of ~3.48 m, is employed for scanning the litter-covered surface (see Figure 1). The PTZ camera provides extensive area coverage and the ability to zoom in for finer details using a single-color camera. This camera possesses three degrees of freedom, including pan, tilt and zoom. Every few minutes, the PTZ camera is directed to a predefined location, scanning the entire litter-covered surface in a zigzag-like motion pattern. Once the camera reaches a new location, a still image is captured and then transferred to the image processing model where segmentation and classification tasks are performed. Images are initially saved at 1796×1009 px resolution and cropped as required.

### 3.1. Image Segmentation

Image segmentation is a very important technique used to separate and classify individual objects in an image by assigning each pixel to a class. In the early stages, the most common segmentation methods were thresholding, histogram-based clustering and k-means clustering, but over the years several advanced deep learning algorithms have been developed that effectively facilitate this task.

One prominent example is U-Net, which was originally developed for medical image segmentation and is one of the first deep learning models specifically designed for segmentation tasks [31]. Moreover, the U-Net structure is widely employed in various Generative Adversarial Network (GAN) variations, including the Pix2Pix generator. The architecture of the model is relatively straightforward, comprising of an encoder responsible for downsampling and a decoder responsible for upsampling [32]. Additionally, the presence of skip connections further enhances the model’s structure.

Mask R-CNN is an advanced deep neural network utilized for image segmentation, known for its exceptional performance [33]. With Mask-RCNN, it becomes possible to automatically generate masks on a pixel level for objects present in an image. This capability enables the precise separation of foreground objects from the background. Mask R-CNN was developed as an extension of Faster R-CNN [34], a renowned object detection model. While Faster R-CNN focuses on generating two outputs for each potential object, namely a class label and a bounding-box offset, Mask R-CNN introduces an additional branch solely dedicated to producing object masks. The inclusion of this supplementary mask output is distinct from the class and bounding-box outputs as it necessitates capturing a much finer spatial representation of an object. The fundamental component of Mask RCNN is the precise alignment of pixels, a crucial element missing in Fast/Faster R-CNN models [33]. Mask-RCNN follows a two-stage process similar to Fast/Faster R-CNN, with an identical initial stage involving a Region Proposal Network (RPN). However, in the second stage, alongside predicting class labels and box offsets, Mask RCNN additionally generates a binary mask for each Region of Interest (RoI) [35]. This approach deviates from many recent systems that rely on mask predictions for classification purposes. Furthermore, the mask branch of Mask RCNN introduces minimal computational overhead, enabling a fast system and facilitating rapid experimentation.

The K-means clustering algorithm is an unsupervised technique which can be employed to separate the region of interest from the background [36]. This becomes highly valuable in situations where unlabeled data are utilized, experts are unavailable for data annotation or when searching for anomalies. The algorithm’s objective is to identify distinct clusters within the data based on their similarity. Applying this algorithm for dropping segmentation could be valuable and serves as both additional information and a cautionary factor, triggering disease identification procedures. Typically, the background value is set to 0 and the desired color spectrum is assigned to the object of interest. Several studies have indicated that K-means-based image segmentation using the Lab color method is more proficient in differentiating object features compared to RGB [37,38]. In our study, we applied the K-means algorithm to each image, following the sequential steps specified in Table 1.

**Figure 2:**
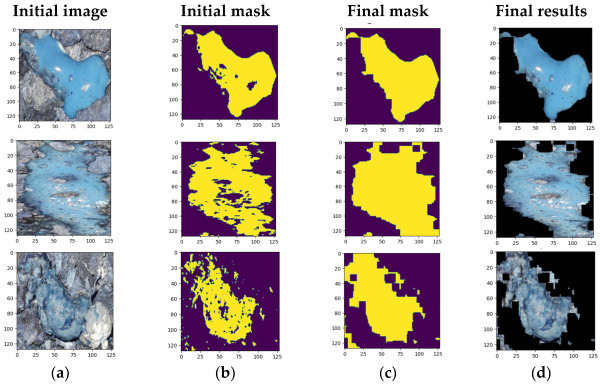
Examples of K-means segmentation results for poultry droppings: (**a**) original image; (**b**) initial; (**c**) final mask; (**d**) final result.

Figure 1 illustrates three examples of poultry dropping segmentations using the K-means algorithm, with bounding boxes applied to crop specific regions of interest within the whole images. Figure 2 shows the sequence from (a) to (d), including the original image, the initially created mask (steps 1 to 3), the final mask (step 7) and the resulting color result. 

### 3.2. Image Classification

Many other deep learning algorithms have gained popularity in image classification. Each algorithm has unique strengths and may be superior depending on the specific task, the dataset and the available computing resources. In our research, we have implemented three different models, namely Resnet101, modified VGG-16 and YOLOv5.

Residual Neural Networks (ResNets are a family of deep learning models that have been widely used for various computer vision tasks, including image classification [39]. The ResNet family comprises various variations, including well-known models like ResNet50, ResNet101, ResNet152 and more. Each model presents a unique trade-off between depth and computational complexity, but, in general, ResNets were developed to tackle the challenge of vanishing gradients in deep neural networks. They introduced residual connections, which enable the training of deep networks by effectively propagating gradients through skip connections. As a result, different ResNets models have demonstrated outstanding performance on a wide range of image classification tasks [40,41,42,43,44].

**Figure 3:**
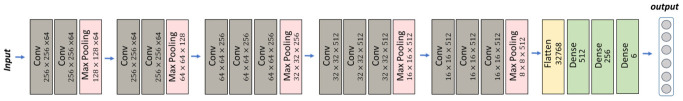
VGG-16 model for classification task.

The VGGNet architecture is renowned for its simplicity and uniformity. It is composed of a sequence of convolutional layers with small receptive fields, followed by fully connected layers. VGG-16, as the name suggests, refers to the specific variant of VGGNet that contains 16 layers with learnable weights [45]. Despite its relatively large size, with around 138 million parameters, VGG-16 remains a popular choice for image classification tasks [46,47,48] due to its simplicity, ease of use and compatibility with transfer learning techniques. We have implemented a modified VGG-16 architecture in our model, utilizing different filters, kernel sizes and dense layer unit numbers compared to the original, with the aim of potentially improving accuracy for our task (see Figure 3).

YOLO (You Only Look Once) is a well-known and widely used set of object detection models in computer vision. Initially proposed in 2016, YOLO combines object classification and localization within a single network, making it a popular choice for object detection tasks [49]. Over time, YOLO has experienced numerous advancements, evolving from its initial version to subsequent iterations, including version 8. Each version includes improvements to the model architecture and features to improve object detection accuracy and speed. In our research, we conducted experiments using YOLOv5 [50], which includes four distinct models, each with different structures consisting of the Input, Backbone, Neck and Prediction components, enabling efficient and accurate object detection [51,52,53].

### 3.3. Proposed Model

**Figure 4:**
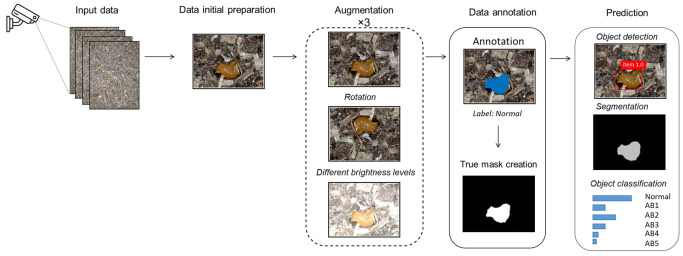
Schematic diagram of a decision-making system for dropping segmentation and classification.

In this study, we collected data from a specific poultry farm in Lithuania using Ezviz C3X cameras and a decision-making model based on deep learning techniques (see Figure 4). This model primarily processes the data as there are images where it is difficult to see poultry droppings due to poor image quality, blurring or similar. Due to the small amount of data, data augmentation was performed using several different techniques such as rotation and brightness level differentiation. The data were manually annotated by a veterinary expert who divided the data into six separate classes. Furthermore, the expert provided the necessary labeling (using PixelAnnotationTool, version 1.4.0) for the segmentation and identification task. Once the object is detected, it undergoes classification into six distinct classes. The final class is determined by identifying the dominant class among them. The object detection threshold is 0.5, i.e., objects with confidence values greater than or equal to 0.5 are considered as detected, while objects with confidence values less than 0.5 are excluded from the final output.

### 3.4. Accuracy Evaluation Metrics

The F1-score is a widely used metric for evaluating the performance of a classification model, especially in scenarios where we want to balance both precision and recall. Hence, these three metrics (precision, recall, and F1-score) were computed to evaluate the automatic classification of droppings images:

(1) $$Precision=\frac{TP}{TP+FP}$$

(2) $$Recall=\frac{TP}{TP+FN}$$

(3) $$F1-score=\frac{2\times Precision\times Recall}{Precision+Recall}$$

where TP (true positive)—the number of positive class samples correctly classified by a model; FP (false positive) the number of samples in the negative class that the model (incorrectly) assigned to the positive class; FN (false negative) the number of samples in the positive class that were (incorrectly) assigned by the model to the negative class.

In the case of multiple classes, the F1-score for each class is calculated using the one-against-one (OvR) method. In this method, the performance of each class is determined separately, as if a separate classifier were used for each class. But, instead of assigning several F1-scores to each class, it is more appropriate to derive an average and obtain a single value to describe the overall performance. There are three types of averaging methods commonly used to calculate F1-scores for multi-class classification, but weighted averaging is the most appropriate for unbalanced data.

Weighted averaging involves calculating the F1-score for each class separately and then taking the weighted average of these individual scores. The weight assigned to each class is proportional to the number of samples in that class. In this case, the F1 result is biased towards the larger classes.

(4) $${Weighted}_{avg}F1\;Score=\sum_{i=1}^{n}w_{i}{\times F1\;Score}_{i}$$

(5) $$w_{i}=\frac{k_{i}}{N}$$

where N—total number of samples, number of samples $k_{i}$ in class i.

Intersection over Union (IoU) is a widely utilized evaluation metric in the field of computer vision, specifically for the segmentation task. IoU measures the overlap between a ground truth bounding box B and a predicted bounding box A. To calculate the IoU, you need to determine the intersection area (common area) and the union area of the two mentioned boxes:

(6) $$IoU=\frac{Area\;of\;Overlap}{Area\;of\;Union}=\frac{\left|A\cap B\right|}{\left|A\cup B\right|}=\frac{TP}{\left(TP+FP+FN\right)}$$

The Dice coefficient is very similar to the IoU; however, it is calculated as twice the intersection of the two sets divided by the sum of their sizes. The Dice coefficient ranges from 0 to 1, where a value of 1 indicates a perfect overlap or segmentation match, while a value of 0 represents no overlap.

(7) $$Dice=\frac{2*\left|A\cap B\right|}{\left|A\right|+\left|B\right|}$$

## 4. Data

The collected image dataset consists of 487 pictures, and each class was labelled in a specific color mask class, contained separate objects can be viewed in Table 2.

**Figure 5:**
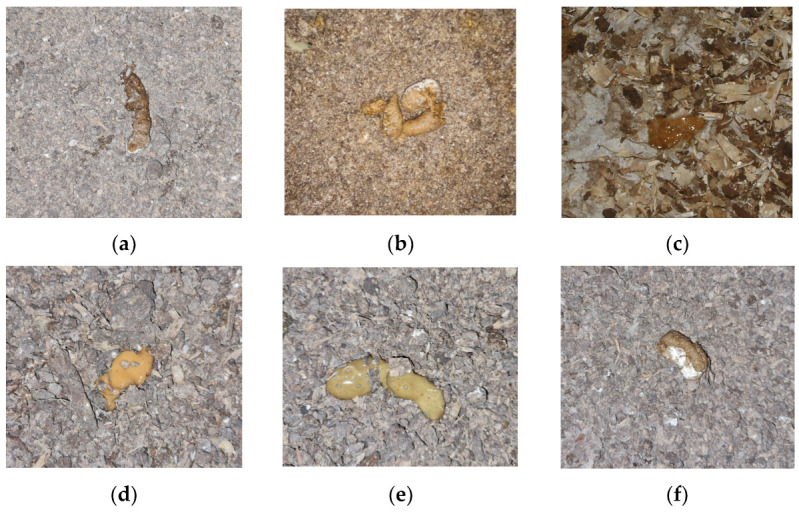
Examples of each of the six class images showing droppings on different types of litter: (**a**) Normal; (**b**) ABN1; (**c**) ABN2; (**d**) ABN3; (**e**) ABN4 and (**f**) ABN5.

Figure 5 displays sample photos representing each class. Normal feces from chickens usually consist of a solid, brown or greyish-brown part with a white, chalky part made up mainly of uric acid from their urinary tract (Figure 5a). If there are small changes in the shape and texture of the faces, this indicates a minor abnormality (Figure 5b). A marginal change in dropping form and structure may suggest mild health issues. Feces may be loose, irregularly shaped and discolored, possibly due to minor bacterial enteritis where inflammation in the intestines disrupts normal gut function, or early stage dysbiosis. Such abnormalities are usually an early warning sign of possible health problems that may require further investigation and intervention. Gas frothiness or bubbly droppings often indicates an underlying infection or disease (Figure 5c). This can be due to conditions such as necrotic enteritis, a disease caused by the bacterium Clostridium perfringens which can produce gas in the intestinal tract. When such feces are detected, a prompt response is needed as they can lead to a reduction in productivity and even increased mortality when untreated. 

If there is a significant amount of moisture and discoloration in the feces (see Figure 5d), it usually means diarrhea, which can occur for a variety of reasons because diarrhea can be caused by a bacterial or parasitic disease. Such diseases can lead to malabsorption syndrome, weight loss and reduced growth, and should be treated urgently in order to avoid serious consequences. Moreover, the combination of high moisture and gas frothiness in droppings may imply a severe digestive disturbance or disease (Figure 5e). Visible undigested feed particles in the droppings is generally a clear sign of malabsorption syndrome. In this case, the chicken’s digestive system is not adequately processing the consumed feed, resulting in particles passing through the system without being digested. This may be due to inflammation in the intestines from bacterial enteritis or damage to the intestinal wall from coccidiosis. It may also be due to a change in the diet that the bird’s digestive system cannot handle. This signifies significant health concerns as malabsorption can lead to nutrient deficiencies, weight loss and reduced growth rates. The detection of undigested food particles in the droppings (Figure 5f) may indicate the presence of malabsorption syndrome. This is a major health concern as malabsorption can lead to nutrient deficiencies, weight loss and stunted growth. These six categories can be visually identified and each necessitates a unique response rate and often involves distinct treatment measures or medications. All of these possible causes and diseases are assumptions that need to be verified since diagnosis is commonly made by directly or indirectly identifying either the agent or serologically.

## 5. Experimental Results

### 5.1. Segmentation Results

**Figure 6:**
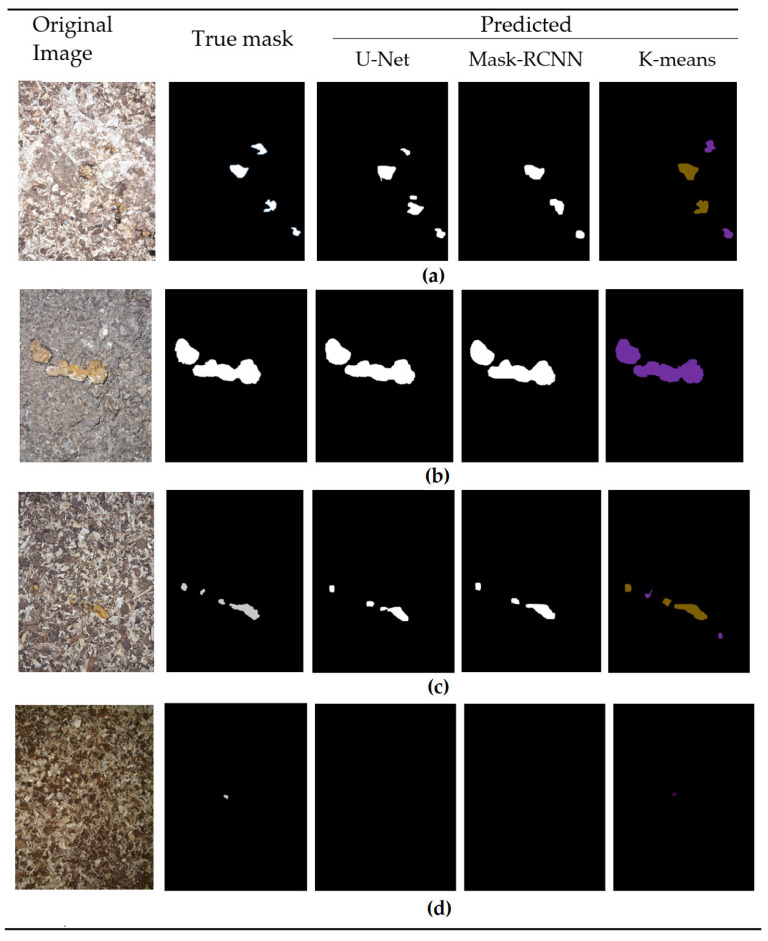
Instances of Segmentation results for 4 different classes: (**a**) Normal; (**b**) ABN1, (**c**) ABN4 and (**d**) ABN3.

To investigate different segmentation methods, we implemented three different algorithms: K-means, U-Net and Mask-RCNN architectures. The images of the segmentation results (predicted masks), including the predicted masks, original image and true mask, are provided below in Figure 6. In the case of the K-means algorithm, we set the number of clusters (K) to 3. As illustrated in Figure 6, the algorithm successfully predicts the masks with a high level of accuracy, achieving an average dice coefficient value of 0.8875 across all six classes. Comparatively, the other two algorithms displayed slightly lower results. The Mask-RCNN algorithm obtained an average dice coefficient of 0.8530, while the U-Net algorithm achieved 0.8746. 

The most complex scenarios occur when there is a small piece of feces on the litter, accompanied by a variety of objects with a similar color and shape characteristics to the feces. It is therefore challenging to detect such a small object that is almost indistinguishable (see Figure 6d).

Table 3 provides the average Dice coefficient values obtained from the segmentation for all six classes of droppings. Despite the K-means model having the highest average Dice coefficient, the U-net model had a slight advantage in two of the six classes. Specifically, in the Normal class, the U-net model achieved a 0.18% higher segmentation accuracy compared to K-means, while for the ABN4 class it achieved 0.93% higher accuracy. The Mask-RCNN model yielded the lowest dice coefficient values for all classes. The K-means algorithm demonstrates its pronounced advantage in the ABN3 class, exhibiting a 7.45% higher dice coefficient value compared to U-Net and a 10.04% higher dice coefficient value compared to Mask-RCNN.

**Figure 7:**
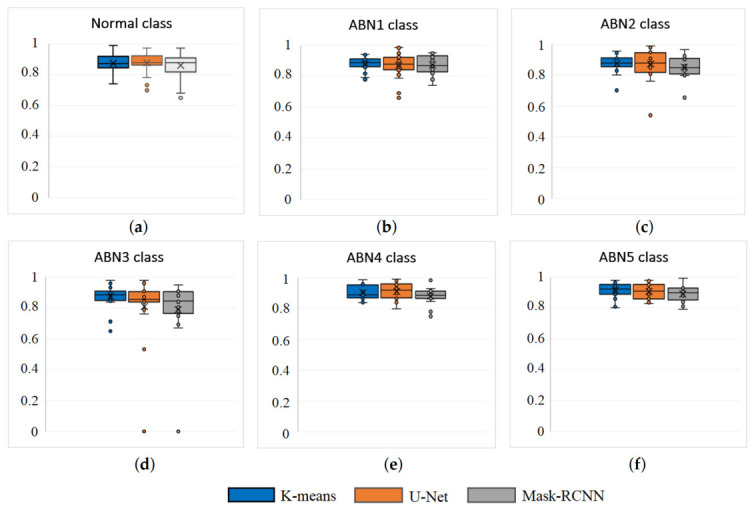
Instances of Segmentation results for 4 different classes: (**a**) Normal; (**b**) ABN1, (**c**) ABN2 (**d**) ABN3, (**e**) ABN4 and (**f**) ABN5.

The advantage of the K-means model can be explained by the ABN3 class of cases where the size of the object is very small (as shown in Figure 6d), where both the U-Net model and the Mask-RCNN model encounter difficulties in segmentation, resulting in a dice coefficient of 0 (see Figure 7d). The segmentation results of the ABN3 class are distinguished by a larger number of outliers in the dice coefficients, which are also found in the other classes, while the segmentation results for class ABN5 are the most stable and accurate (see Figure 7f).

### 5.2. Classification Results

For the classification task, we have implemented three different models as well and the accuracy results are provided in Table 4. The YOLOv5 model gives the best results with an accuracy of 91.78%. The ResNet-101 model gives slightly worse results with a 91.10% accuracy (<1% difference). And the worst results were obtained with the VGG-16 model with an accuracy of 89.24%. As our data are unbalanced (the largest class has 195 testing data and the smallest has 16), it is appropriate to provide a weighted average of F1 scores as an indicator for evaluation.

**Figure 8:**
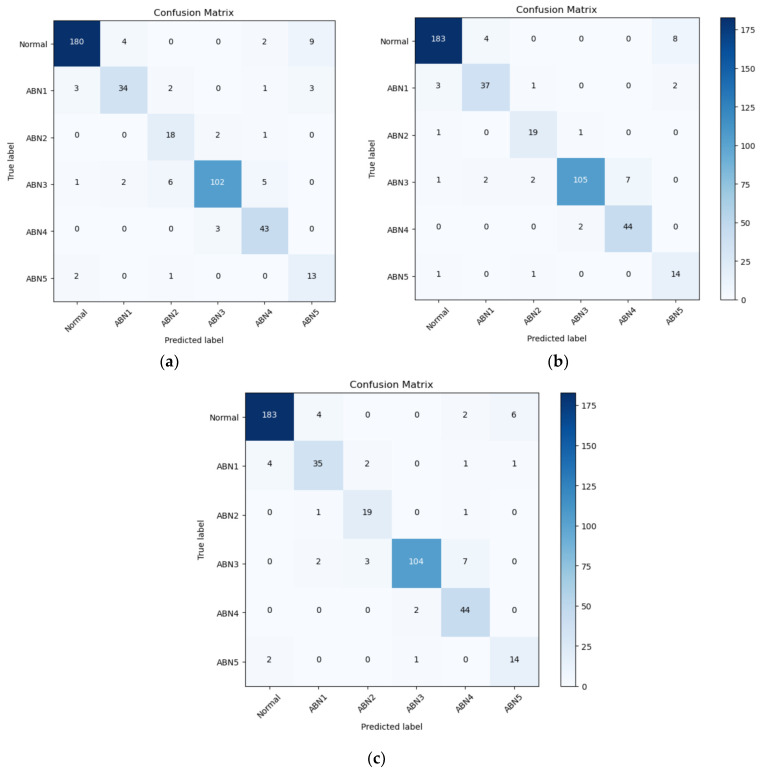
Confusion matrix of three different classification models: (**a**) VGG-16, (**b**) YOLOv5, (**c**) ResNet-101.

Observing the results of confusion matrixes (see Figure 8) we can see that the best classification results are given by the Normal class (with F1-scores of 95.31% for ResNet-101 and YOLOv5 and 94.48% for VGG-16) and the worst by ABN2 and ABN5, but in these classes we have the least data. In the classification process, there is a notable confusion between the classes Normal and ABN5, as well as between ABN3 and ABN4.

## 6. Discussion

**Figure 9:**
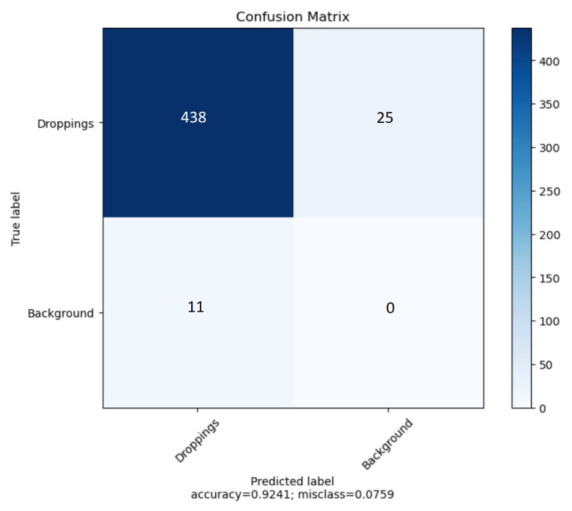
Object detection confusion matrix.

**Figure 10:**
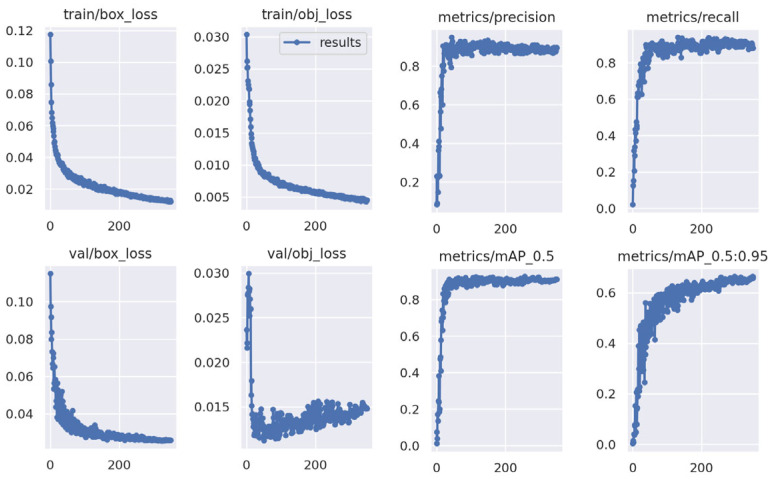
Precision, Recall and mAP plot for YOLOv5 training.

In our study, we have used YOLOv5 for binary object detection, specifically for detecting object class and the background. The confusion matrix is presented in Figure 9 to assess the model’s performance on the task. The classification accuracy (ACC) stands at 0.9241, whereas the F1-score is 0.9605. The algorithm is more likely to fail to detect existing objects than to detect objects that are not actually present in the image. This is usually the case when there are many objects in the photo, usually more than four (see Figure 10).

The YOLOv5m model has been implemented and training was carried out over 350 epochs. Figure 10 presents the training results, which include a precision score of 0.891 recall of 0.892 and mAP (mean Average Precision) of 0.901 and 0.601 for 0.5 IOU and 0.95 IOU, respectively (95%).

**Figure 11:**
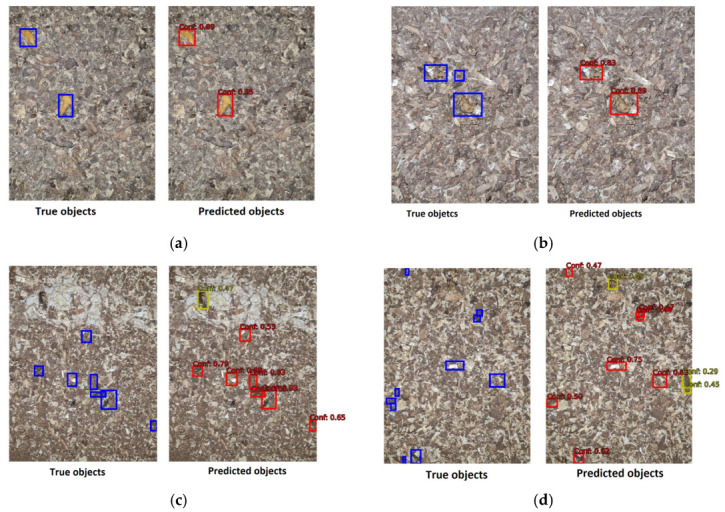
The sample images with true object (left side of image) and predicted objects with confidence level (right side of the image): (**a**) perfect detection with high confidence level; (**b**) two of three object have been detected; (**c**) one object has been incorrectly identified as dropping; (**d**) three objects incorrectly identified as droppings and three are missing.

Figure 11 shows some examples of object detection. It is noted that usually only very small pieces of droppings are missing, which is, in principle, not very significant in a more holistic view of the problem itself. On the other hand, objects that are not detected usually have a low confidence level and, thus, do not pass the threshold value (0.5) and are therefore not evaluated in the final solution. A detailed review of such cases reveals that some of the objects inaccurately detected may actually exist and have been overlooked by an expert, possibly due to human error or because they are very small.

In this research, we also conducted binary classification, categorizing data into two classes: normal and abnormal. As depicted in the results table (see Table 5), such classification approach showed a slight improvement of 2.74%, achieving an accuracy (ACC) of 94.52%. Table 5 also presents the experimental results of other authors’ attempts at classifying droppings into 3–5 categories. However, it is important to note that direct comparison is difficult due to the differences in datasets, data collection conditions (real or laboratory), data quality and the number of dropping classes used for classification. A number of studies have been carried out using open source Kaggle 4-class dataset (Poultry Diseases Detection), which includes images of “Newcastle”, “Salmonella”, “Coccidiosis” and “Healthy” droppings.

Such research aligns with the current global trends in poultry farming and the overarching research concept of poultry gut health. This indicator depends on many factors, such as feed production technology, compound feed composition and structure, oil quality, protein and amino acid content in the feed, fiber and its components, macro and microelements such as calcium, phosphorus, sodium, copper, zinc, selenium and feed additives such as glycerides of medium-length fatty acids, NSP enzymes, etc. It is therefore, reasonable to take into account the effect of the above indicators on the health of the poultry, which will allow a more efficient prediction of the status of poultry health through the utilization of data analytics (e.g., correlations, monitoring of dynamics).

## 7. Conclusions

Within the scope of this study, we have developed a computer vision-based solution with a primary emphasis on the early detection of abnormal poultry droppings through non-invasive droppings analyses. The main objective was to provide an additional visual factor and to enable prompt disease outbreak control and to contribute to optimal bird health management. In our research, the droppings were categorized into six classes, guided by expert veterinary knowledge and visual indicators, which signified specific levels of abnormalities. The conducted experiments highlighted the proficiency of the proposed model in recognizing and categorizing both individual and multiple occurrences of droppings within a single image. The results obtained revealed that the deep learning model achieved a detection accuracy of 92.41% for droppings, even when presented with up to 11 objects. Notably, it was observed that only small pieces of droppings were undetected or inaccurately detected, which has little or no impact on the overall decision making.

After evaluating multiple machine learning algorithms for the segmentation and classification tasks, we have created a decision-making system based on the obtained results. The results indicated that the K-means algorithm outperformed U-Net and Mask-RCNN in the segmentation task, achieving the highest accuracy of a 0.88 Dice coefficient. Among architectures such as VGG-16 and ResNet-101, YOLOv5 demonstrated superior performance, achieving the highest accuracy of ACC = 91.78% for classification task. However, it should be noted that the dataset is unbalanced and the lowest accuracies are obtained with the anomaly classes with the least data. Therefore, it is likely that in the future the collection of more images with these classes will not only increase the accuracy but also allow for more detailed studies to be carried out to identify more class-specific features to assess correlations with diet, poultry age, etc.

## Figures and Tables

**Table 1:** Pre-processing steps in image feature extraction using K-means algorithm.

No.	Steps:
1.	Transform the image: Convert an RGB image into the HSI and the Lab color space;
2.	Apply OTSU thresholding: Use the OTSU algorithm to create a binary image, differentiating the objects from the background.
3.	Perform thresholded image corrections: apply erosion and dilation operations.
4.	Invert the threshold: consider the black part as the background of the image.
5.	Extract edges/contours: utilize Suzuki’s algorithm to extract the edges or contours from the image.
6.	Filter out smaller contours: remove smaller contours from the extracted edges.
7.	Features extraction:
7.1	Compute the convex hull and calculate the perimeter and area of the hull.
7.2	Obtain morphological information: length, width, perimeter, area and bounding points.
7.3	Generate distance maps.

**Table 2:** The dataset consisted of six distinct classes of droppings.

Classes	Description	Initial Amount of Images	Amount of Images after Augmentation
Normal	Normal droppings, good form and structure, low moisture amount	216	648
ABN1	Abnormalities: marginal change of droppings form and structure	47	141
ABN2	Abnormalities: gas frothiness	23	69
ABN3	Abnormalities: high moisture amount	130	390
ABN4	Abnormalities: high moisture amount and gas frothiness	53	159
ABN5	Abnormalities: undigested feed particle	18	54
	Total	487	1461

**Table 3:** The Dice coefficient’s average values determined from the segmentation test.

Algorithm	Class						Average
	Normal	ABN1	ABN2	ABN3	ABN4	ABN5	
Mask_RCNN	0.85993	0.87388	0.85232	0.79055	0.87945	0.89119	0.8579
U-Net	0.87674	0.87404	0.87260	0.81130	0.91259	0.90896	0.8760
K-means	0.87518	0.87947	0.87820	0.87408	0.90414	0.91393	0.8875

**Table 4:** Accuracy values of classification models.

Model	Precision	Recall	Accuracy	Macro-F1	Weighted Average F1
VGG-16	79.74%	86.62%	89.24%	82.34%	89.67%
ResNet-101	83.486	88.89%	91.10%	86.03%	91.28%
Yolov5	84.55%	90.54%	91.78%	86.96%	92.03%

**Table 5:** Accuracy values of classification models.

Classes	References	Dataset Size	Algorithm/Model	Classification Results (Metric)
(1) Health (2) Coccidiosis (3) Salmonella	Degu, M.Z. et al. [28]	10,500	ResNet50	98.70% (ACC)
	Mbelwa, H. et al. [16]	1590	XceptionNet	94.00% (ACC)
			CNN	93.67% (ACC)
			VGG 16	89.33% (ACC)
(1) Newcastle (2) Salmonella (3) Coccidiosis (4) Healthy	Liu, X. et al. [54]	8067	PoultryNet	97.77% (ACC)
	Chen, X. et al. [55]	8067	ResNeXt50-3A	97.40% (ACC)
	Machuve, D. et al. [23]	1255	InceptionV3	95.45% (ACC)
			MobileNetV2	98.02% (ACC)
			Xception	98.24% (ACC)
(1) Normal (2) Abnormal shape (3) Abnormal color (4) Abnormal water content (5) Abnormal shape and water	Jintao Wang [27]	7637	Yolo V3	88.70% (Recall)
			Faster R-CNN	99.10% (Recall)
(1) Normal (2) Abnormal	Our proposed model	1461	K-means + YOLO V5	94.52% (ACC)
(1) Normal (2) Abnormal with minor changes (3) Abnormal with gas foaming (4) Abnormal with moisture content (5) Abnormal with undigested feed			K-means + YOLO V5	91.78% (ACC)

## Data Availability

The data presented in this study are available from the corresponding author upon request.
